# HTLV-1 associated primary cutaneous tumoral ATLL in a Floridian without travel to an endemic area

**DOI:** 10.1016/j.jdcr.2025.09.013

**Published:** 2025-09-24

**Authors:** Priya Patel Housley, Caitlin G. Purvis, Pooja R. Gurram, Kiran Motaparthi

**Affiliations:** a College of Medicine, University of Florida, Gainesville, Florida; b Department of Dermatology, College of Medicine, University of Florida, Gainesville, Florida; c Department of Infectious Diseases & Global Medicine, University of Florida, Gainesville, Florida

**Keywords:** adult T-cell leukemia/lymphoma (ATLL), cutaneous lymphomadisseminated strongyloidiasis, Human T-cell lymphotropic virus type 1 (HTLV-1), strongyloides stercoralis

## Introduction

Human T-cell lymphotropic virus type 1 (HTLV-1) is a retrovirus that integrates into CD4+ T cells and can silently persist in hosts. Most cases remain asymptomatic; however, 5% of infected individuals develop adult T-cell leukemia/lymphoma (ATLL), an aggressive and often fatal malignancy.^1^ HTLV-1 interferes with host immunity, increasing the prevalence of hyperinfection with *Strongyloides stercoralis*, a parasitic nematode.^2^ As a result, individuals are susceptible to disseminated strongyloidiasis (DS); conversely, *Strongyloides* infection is associated with elevated HTLV-1 proviral load and increased risk of leukemogenesis.^2^

Although HTLV-1 is endemic to Japan and the Caribbean, sporadic cases have been reported in southeastern United States.^3^ We present a diagnostically challenging case of a Floridian woman with *Strongyloides stercoralis* infection, whose cutaneous findings led to diagnosis of underlying HTLV-1 associated ATLL.

## Case presentation

A 57-year-old woman with a past medical history of end-stage renal disease (ESRD) on hemodialysis and no travel history was transferred to our institution in April 2024 for concern of encephalopathy and multiorgan failure. One month prior, she was admitted to an outside facility for small bowel obstruction with enterocolitis, during which an intestinal biopsy demonstrated *Strongyloides stercoralis*. She was treated with ivermectin and later developed *vancomycin-resistant Enterococcus faecium* (VRE) bacteremia treated with linezolid.

Upon arrival, lumbar puncture and cerebrospinal fluid analysis revealed VRE meningitis. She was treated with a prolonged course of linezolid and ivermectin due to concern for *Strongyloides* hyperinfection. Over the following months, she was readmitted several times for recurrent VRE meningitis, cerebritis, and sepsis, which required treatment with daptomycin, tigecycline, and long-term omadacycline for suppression.

**Fig 1:**
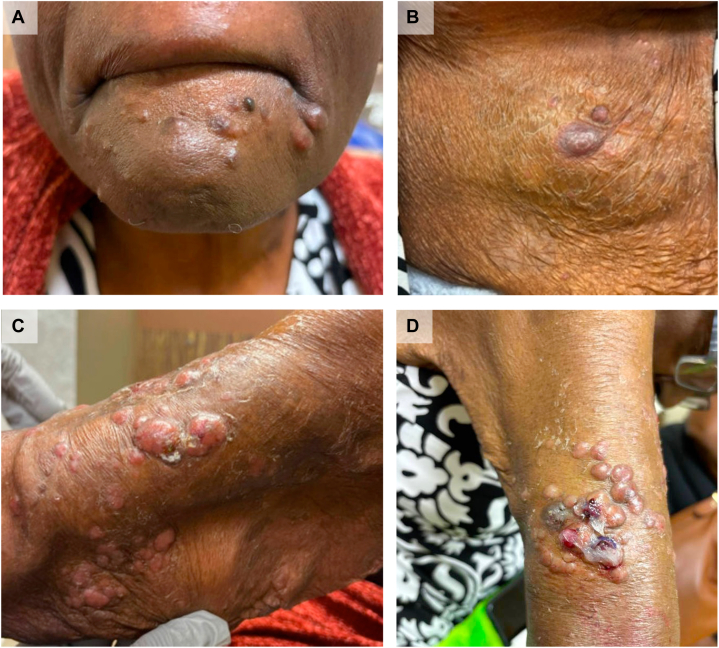
Cutaneous findings in patient with HTLV-1 associated adult T-cell leukemia/lymphoma with *Strongyloides stercoralis* co-infection. **A,** Smooth *pink papules* and nodules on the chin. **B,***Pink hyperpigmented nodule* with dry scale on the back. **C,** Crusted *pink nodules* on medial arm. **D,** Crusted violaceous and erythematous papules on extensor surface near elbow.

In October 2024, she was admitted for a right upper extremity abscess near a hemodialysis fistula. Culture from aspirate of the abscess grew *Trichophyton,* which was initially thought to be a contaminant. She developed progressive, pruritic nodular skin lesions on the face, trunk, extremities, buttocks, and thighs (Fig 1). Despite treatment with fluconazole and later terbinafine, the lesions progressed in size and number. Repeat cultures grew *Trichophyton rubrum*. At this point, given her extensive infectious history along with ESRD, concern for an underlying immunodeficiency was raised. As a part of immunodeficiency work up, she was found to have an elevated CD4:CD8 ratio of 8.32 (range: 0.86-5.00).

**Fig 2:**
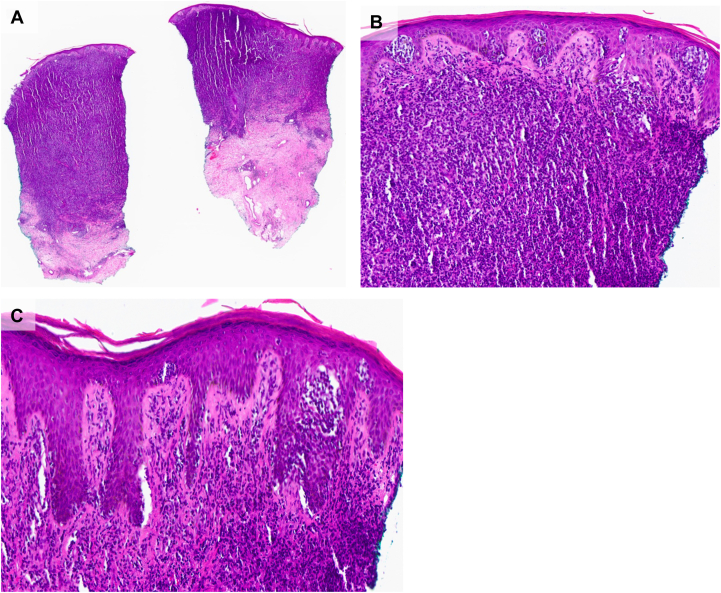
H&E histopathology of HTLV-1 associated ATLL. **A,** Diffuse and dense lymphoid infiltrate extending into the deep dermis (10×). **B,** Numerous collections of atypical lymphocytes in the epidermis with a dense, band-like dermal lymphoid infiltrate (100×). **C,** Psoriasiform acanthosis with minimal spongiosis and an increased number of lymphocytes at the dermoepidermal junction with epidermotropism (200×).

**Fig 3:**
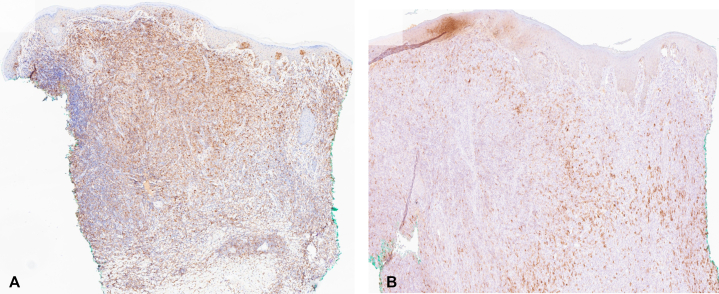
CD25 and CD30 immunohistochemical stains of HTLV-1 associated ATLL. **A,** CD25 stain highlights atypical lymphocytes in dermis and epidermis (30×). **B,** CD30 stain displaying focal expression (100×).

**Fig 4:**
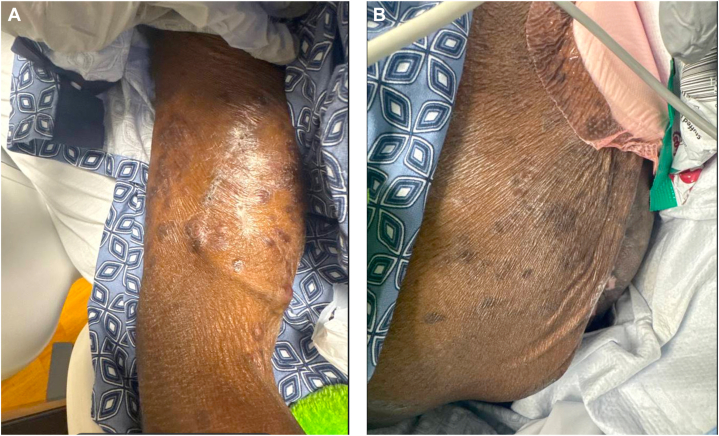
Cutaneous findings in patient with HTLV-1 associated adult T-cell leukemia/lymphoma with *Strongyloides stercoralis* co-infection after treatment with brentuximab vedotin. **(A, B)** Clinical improvement in nodules after treatment.

In December 2024, given the constellation of findings (hyperinfection with *Strongyloides*, recurrent VRE meningitis, cutaneous *Trichophyton* infection, and high CD4:CD8 ratio), HTLV-1 associated lymphoma was suspected. A punch biopsy and histopathological evaluation of the skin nodules confirmed ATLL (Figs 2 and 3). HTLV-1 serology was positive with a whole blood quantitative PCR of 1,900,000 copies/mL. CT imaging revealed splenomegaly, multiple lung nodules, and an enlarged left axillary lymph node. The patient was diagnosed with HTLV-1 associated ATLL and is under the care of oncology, infectious disease, nephrology, and dermatology. She has received 1 cycle of brentuximab vedotin with complete resolution of skin nodules (Fig 4) and has shown improvement with chemotherapy.

## Discussion

HTLV-1, a retrovirus that integrates into CD4+ T cells, remains silent in most infected individuals. However, 5% of those infected develop ATLL, an aggressive malignancy with a poor prognosis and overall survival of less than 1 year.^1^ Although HTLV-1 is endemic to regions such as Japan and the Caribbean, this Floridian patient had no travel history.^3^ Her diagnosis was unexpected and highlights consideration in non-endemic regions.

There has been a documented association between HTLV-1 and *Strongyloides stercoralis* infection.^2^ The virus promotes immune dysfunction by altering the ratio of Th1/Th2 mediated cytokines and impairs IgE mediated immune responses, subsequently predisposing individuals to hyperinfection and disseminated strongyloidiasis.^2^ Conversely, chronic strongyloidiasis has been shown to increase the proviral load of HTLV-1 and promote leukemogenesis.^2^^,^^4^ Oncogenesis is driven by pathways including persistent NF-kB activation and clonal proliferation of infected T cells.^1^^,^^5^

Diagnostic testing for *Strongyloides* in patients with HTLV-1 co-infection remains challenging. Immunoassays for *Strongyloides*, notably ELISAs, have been reported to have severe limitations including low sensitivity in patients with HTLV-1 or hematological malignancies.^6^ In terms of serologic testing with co-infection, while 89% of patients with mild strongyloidiasis were observed to have IgE antibodies against *Stongyloides*, IgE antibodies were not detected in those with severe strongyloidiasis.^2^ In this patient, *Stongyloides* infection was confirmed during a prior hospitalization, however, a month later, subsequent serology and stool testing were negative. This is consistent with previous reports during which standard diagnostics failed to detect *Strongyloides* in patients severely immunocompromised due to HTLV-1.^2^^,^^6^ However, a study found that co-infection did not decrease IgG antibodies against *Strongyloides* antigen and may assist in diagnosis.^2^ Despite the negative results, this patient was re-treated with ivermectin due to concern for hyperinfection in addition to anti-VRE antibiotics. While only 1% of individuals with mild strongyloidiasis have positive serological testing for HTLV-1, 75% of the patients with severe strongyloidiasis have positive serology for HTLV-1.^2^ Clinical suspicion for ATLL should be high in patients with severe strongyloidiasis and cutaneous manifestations that are refractory to treatment.

Cutaneous manifestations are common in ATLL, particularly the primary cutaneous tumoral (PCT) and smoldering variants. In the United States, 67% of patients presented with skin lesions at the time of diagnosis; morphologies included plaques, papular eruptions, erythroderma, and nodulotumoral lesions.^4^ Cutaneous findings may mimic dermatophytosis, eczema, or bacterial infections, leading to a delay in diagnosis. Additionally, cutaneous involvement has been shown to be a prognostic indicator; patients with the PCT variant had a median survival of 20 months, while those with the smoldering type had a median survival of 109 months. In a multivariable analysis, a study found that skin involvement was related to a worse outcome, regardless of clinical form and presence of lymphadenopathy.^4^ In our patient, the progressive nodules were initially thought to be disseminated *Trichophyton* infection due to prior culture results. However, her condition worsened despite antifungal treatment, raised suspicion, and prompted punch biopsy revealing a cutaneous T-cell lymphoma (CTCL). This aligns with previous descriptions of skin manifestations as an early and prominent sign of ATLL. As the histopathology of ATLL can resemble other CTCLs (including mycosis fungoides and CD30+ lymphoproliferative disorders), establishing the correct diagnosis requires knowledge of patients’ HTLV-1 status.

## Conflicts of interest

None disclosed.
